# Risk for Invasive Streptococcal Infections among Adults Experiencing Homelessness, Anchorage, Alaska, USA, 2002–2015

**DOI:** 10.3201/eid2510.181408

**Published:** 2019-10

**Authors:** Emily Mosites, Tammy Zulz, Dana Bruden, Leisha Nolen, Anna Frick, Louisa Castrodale, Joseph McLaughlin, Chris Van Beneden, Thomas W. Hennessy, Michael G. Bruce

**Affiliations:** Centers for Disease Control and Prevention, Anchorage, Alaska, USA (E. Mosites, T. Zulz, D. Bruden, L. Nolen, T.W. Hennessy, M.G. Bruce);; Alaska Department of Health and Social Services, Anchorage (A. Frick, L. Castrodale, J. McLaughlin);; Centers for Disease Control and Prevention, Atlanta, Georgia, USA (C. Van Beneden)

**Keywords:** homeless persons, homelessness, streptococci, bacteria, group A Streptococcus, group B Streptococcus, Streptococcus pneumoniae, Anchorage, Alaska, United States

## Abstract

The risk for invasive streptococcal infection has not been clearly quantified among persons experiencing homelessness (PEH). We compared the incidence of detected cases of invasive group A *Streptococcus* infection, group B *Streptococcus* infection, and *Streptococcus pneumoniae* (pneumococcal) infection among PEH with that among the general population in Anchorage, Alaska, USA, during 2002–2015. We used data from the Centers for Disease Control and Prevention’s Arctic Investigations Program surveillance system, the US Census, and the Anchorage Point-in-Time count (a yearly census of PEH). We detected a disproportionately high incidence of invasive streptococcal disease in Anchorage among PEH. Compared with the general population, PEH were 53.3 times as likely to have invasive group A *Streptococcus* infection, 6.9 times as likely to have invasive group B *Streptococcus* infection, and 36.3 times as likely to have invasive pneumococcal infection. Infection control in shelters, pneumococcal vaccination, and infection monitoring could help protect the health of this vulnerable group.

In 2017, the number of persons experiencing homelessness (PEH) in the United States increased for the first time in 7 years to >550,000 persons, coinciding with high-profile outbreaks of infectious diseases such as hepatitis A, shigellosis, and invasive group A *Streptococcus* (GAS) infection among PEH (*1*–*4*). PEH experience unmanaged chronic disease, undernutrition, substance abuse, mental health disorders, crowding, exposure to weather, and limited access to hygiene resources, all of which can increase their risk for infectious disease (*5*,*6*). In high-income countries, baseline tuberculosis prevalence has been estimated to be 22–461 times higher, hepatitis C prevalence 4–70 times higher, and HIV prevalence up to 77 times higher among PEH than among the general population (*7*).

Severe manifestations of invasive streptococcal infections include pneumonia, meningitis, sepsis, cellulitis, and necrotizing fasciitis. Although PEH could be at higher risk for invasive streptococcal infection, only a few outbreaks among PEH were reported before 2015. Investigators in France described a pneumonia outbreak among homeless men during 1989–1991 caused by *Streptococcus pneumoniae* (pneumococcus) (*8*) and 2 invasive GAS outbreaks among PEH in 2009 (*emm* type 44) and 2010 (*emm* type 83) (*9*,*10*). During 2005–2009, an epidemic of invasive pneumococcal disease (serotype 5) was described in the homeless population in western Canada (*11*), and during 2009–2011, an outbreak of invasive pneumococcal disease (serotype 12F) among PEH was reported in Winnipeg, Manitoba, Canada (*12*). Starting in 2015, invasive GAS infections have emerged as a larger problem among PEH than previously recognized, as outbreaks began to be reported in the United States, Canada, and England (*3*,*13*,*14*).

The baseline risk for invasive streptococcal disease has rarely been quantified in the population experiencing homelessness. A case–control study of invasive GAS infection in Barcelona, Spain, during 1998–2003 among persons who used intravenous drugs showed that those with invasive GAS soft-tissue infections were 4 times more likely to be homeless than those without GAS infections (*15*). During 2002–2006, the incidence of invasive pneumococcal disease among PEH in Toronto, Ontario, Canada, was estimated to be 30 times higher than that among the general population (273 vs. 9 infections/100,000 persons/y) (*16*). 

To our knowledge, an estimate of the baseline relative risk for invasive streptococcal infections among PEH in the United States has not been reported. By using data from Alaska’s population-based laboratory surveillance system for invasive bacterial disease, US Census data, and the Anchorage Point-in-Time count, we estimated the baseline risk for invasive disease caused by GAS, group B *Streptococcus* (GBS), and pneumococcus among adults (age >18 years) experiencing homelessness in Anchorage, Alaska, during 2002–2015.

## Methods

### Data Sources

In Alaska, invasive streptococcal disease cases (including pneumococcal, GAS, and GBS cases) are reportable to the Alaska Division of Public Health. In collaboration with the State of Alaska, the Centers for Disease Control and Prevention (CDC) Arctic Investigations Program (part of the National Center for Emerging and Zoonotic Diseases, Division of Preparedness and Emerging Infections) conducts statewide, population-based, and laboratory-based surveillance for invasive infections caused by these pathogens (*17*–*19*). Participating laboratories send sterile site bacterial isolates to the Arctic Investigations Program for confirmatory testing, antimicrobial-susceptibility testing, and molecular typing (*emm*-typing for GAS and serotyping for pneumococcus). Confirmed cases of invasive infection are defined as the isolation of the pathogen from a normally sterile body site (e.g., blood), isolation of GAS from a nonsterile site in persons with necrotizing fasciitis or streptococcal toxic shock syndrome, or isolation of GBS from a nonsterile site in the case of fetal demise. Standardized chart reviews are conducted on all confirmed cases, including information on demographics, associated diagnoses, alcohol abuse, injection drug use, and underlying conditions. Because many cases were detected through blood culture, bacteremia was often present in addition to other diagnoses. We report diagnosis of bacteremia as bacteremia alone, without other diagnoses. Information on homelessness was routinely collected from the medical record beginning in 2002. This routine public health surveillance is considered nonresearch by the CDC and Alaska area institutional review boards.

We did not include data from 2016 in this analysis because a large outbreak of invasive GAS infection occurred among the homeless population in Anchorage beginning in February 2016 (*3*). We also limited our study to cases among adults (age >18 years) because only 2 cases of invasive streptococcal disease were detected in children experiencing homelessness over the study period.

For the years 2005–2015, we used the general Anchorage adult population data from the US Census and homeless adult population data from the Anchorage Point-in-Time count (PIT) (*20*,*21*). PIT is a yearly count of sheltered and unsheltered homeless persons made on a single night in January, as mandated by the US Department of Housing and Urban Development for communities receiving federal funds from the McKinney–Vento Homeless Assistance Grants program (*22*). In these counts, a person is considered homeless if they are spending the night in an emergency shelter or sleeping in a car, tent, or other area considered not suitable for human habitation; persons who are staying with relatives or friends or who are in short-term or transitional housing are not included. Homeless population data were not available for 2002–2004, so we used the mean data from 2005–2015 for those years. We restricted the analysis of case and population data to Anchorage because homeless population data were available for Anchorage but not for other urban centers in Alaska (such as Juneau or Fairbanks). Limited demographic information was available for rate adjustment. Age and sex distributions were used from the US Census, but age information was not available from PIT. We estimated the age distribution of PEH in Anchorage by using data from a survey conducted during a large homeless outreach project called Project Homeless Connect from 2010 (*23*).

### Statistical Methods

Cases of invasive GAS, GBS, and pneumococcal disease were classified as occurring in PEH if “homeless” was checked on the surveillance chart review form. Otherwise, cases were classified as being in persons in the general population. For the purposes of this analysis, the case and population data labeled as general population excluded PEH. We calculated invasive streptococcal infection incidence per 100,000 person-years for PEH and the general population, deriving annual population denominators from PIT for PEH and from the Anchorage census count minus the PIT estimate for the general population. We conducted direct age standardization of the incidence of GAS, GBS, and pneumococcal invasive disease by using the general population census age structure as the standard population. We calculated the incidence rate ratio (IRR) and 95% CIs comparing invasive streptococcal infection incidence in the homeless population to that among the general population by using Poisson exact tests. We also calculated risk differences for each invasive streptococcal infection between PEH and the general population and the percentage of each infection type associated with homelessness. We compared characteristics such as demographics, diagnoses, and coexisting conditions between cases among PEH and the general population by using χ^2^ tests and Fisher exact tests.

## Results

During 2005–2015, PIT counted a mean number of 970 adults (minimum 795, maximum 1,486) in Anchorage who were homeless, either sleeping in a shelter or sleeping outside. The mean general population in Anchorage during this period was 288,921 adults (minimum 264,795, maximum 300,175) who were not experiencing homelessness. The largest age stratum for both PEH and the general population was 31–50 years, but this stratum was larger for PEH (55% among PEH vs. 29% among the general population). From 2002 through 2015, the Arctic Investigations Program surveillance system detected 56 cases of invasive GAS infection, 6 cases of invasive GBS infection, and 84 cases of invasive pneumococcal infection in the adult population experiencing homelessness in Anchorage. Among the general population in Anchorage, the system detected 229 cases of invasive GAS infection, 194 cases of invasive GBS infection, and 457 cases of invasive pneumococcal infection (Table 1).

**Table 1:** Demographic and clinical characteristics of adults with invasive streptococcal infection compared with the general adult population, Anchorage, Alaska, USA, 2002–2015*

Characteristic	Persons experiencing homelessness	General population	p value
Group A *Streptococcus* case-patients	56	229	
Age, y, mean (SD)	51 (11)	54 (19)	0.27
Sex			
M	43 (77)	122 (53)	<0.01
F	13 (23)	107 (47)	
Diagnosis			
Cellulitis	37 (66)	107 (47)	0.01
Pneumonia	11 (20)	43 (19)	0.88
Necrotizing fasciitis	9 (16)	14 (6)	0.01
Bacteremia	4 (7)	46 (20)	0.03
Other conditions			
Diabetes	5 (9)	68 (30)	<0.01
Intravenous drug use	5 (9)	8 (3)	0.14
Alcohol abuse	42 (75)	23 (52)	<0.01
Death during episode	6 (11)	29 (13)	0.69
Group B *Streptococcus* case-patients	6	194	
Age, y, mean (SD)	53 (11)	60 (16)	0.28
Sex			
M	4 (67)	97 (50)	0.68
F	2 (33)	97 (50)	
Diagnosis			
Cellulitis	1 (16)	63 (32)	0.67
Pneumonia	2 (33)	24 (12)	0.13
Necrotizing fasciitis	0	0	NA
Bacteremia	2 (33)	63 (32)	1.00
Other conditions			
Diabetes	0	89 (46)	0.03
Intravenous drug use	0	4 (2)	0.72
Alcohol abuse	5 (83)	21 (11)	<0.01
Death during episode	2 (33)	15(7)	0.08
*S. pneumoniae* case-patients	84	457	
Age, y, mean (SD)	48 (9)	57 (17)	<0.01
Sex			
M	55 (65)	258 (56)	0.124
F	29 (35)	199 (44)	
Diagnosis			
Cellulitis	3 (4)	8 (2)	0.39
Pneumonia	76 (90)	369 (81)	0.03
Necrotizing fasciitis	0 (0)	1 (0)	1.00
Bacteremia	4 (5)	53 (12)	0.08
Other conditions			
Diabetes	6 (7)	85 (19)	0.01
Intravenous drug use	3 (4)	8 (2)	0.39
Alcohol abuse	74 (88)	130 (28)	<0.01
Death during episode	6 (7)	60 (13)	0.15

*Values are no. (%) unless otherwise indicated. General population excludes persons experiencing homelessness. p values based on χ^2^ or Fisher exact test. NA, not applicable.

**Figure 1:**
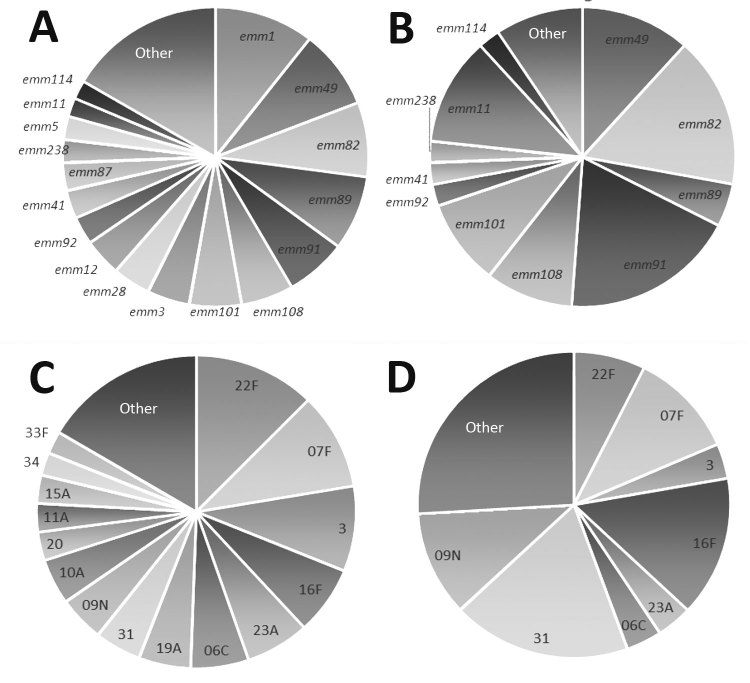
Group A *Streptococcus emm*-type and *Streptococcus pneumoniae* serotype distributions among the general population compared with distributions among persons experiencing homelessness, Alaska, 2002–2015. A) Group A *Streptococcus emm* types among the general population. B) Group A *Streptococcus emm* types among persons experiencing homelessness. C) *S. pneumoniae* serotypes among the general population. D) *S. pneumoniae* serotypes among persons experiencing homelessness. General population excludes persons experiencing homelessness.

PEH with invasive GAS infection were more often male and more likely to be diagnosed with alcohol abuse but less likely to be diagnosed with diabetes than persons in the general Anchorage population with invasive GAS infection (Table 1). PEH with invasive GAS infection were also more likely to have a diagnosis of cellulitis or necrotizing fasciitis than were persons in the general population with invasive GAS infection. The most common *emm* types identified among invasive GAS infection isolates from PEH included *emm*91 (19%), *emm*82 (16%), and *emm*49 (12%), whereas the most common *emm* types identified among persons in the general population were *emm*1 (10%), *emm*49 (9%), *emm*82 (8%), and *emm*89 (8%) (Figure 1). The crude IRR of having a detected case of invasive GAS infection for PEH compared with the general population was 53.7 (95% CI 39.3–72.2), and the age-adjusted IRR was 53.3 (95% CI 46.7–61.0) (Table 2).

**Table 2:** Crude and age-adjusted incidence rates of invasive streptococcal infections among the adult population experiencing homelessness compared with the general adult population, Anchorage, Alaska, USA, 2002–2015*

Disease	Population experiencing homelessness				General population			Crude IRR (95% CI)	Age-adjusted IRR (95% CI)
	No. cases	Person-years	Incidence		No. cases	Person-years	Incidence		
Group A *Streptococcus*	56	13,585	412.2		229	2,983,169	7.7	53.7 (39.3–72.2)	53.3 (46.7–61.0)
Group B *Streptococcus*	6	13,585	44.2		194	2,983,169	6.5	6.8 (2.5–15.0)	6.9 (6.0–8.1)
*Streptococcus pneumoniae*	84	13,585	618.3		457	2,983,169	15.3	40.3 (31.5–51.0)	36.3 (33.0–39.9)

*General population excludes persons experiencing homelessness. Incidence expressed as no. cases/100,000 person-years. IRR, incidence rate ratio.

PEH with invasive GBS were more likely to have alcohol abuse and less likely to have diabetes recorded in their medical record than invasive GBS patients in the general population (Table 1). The crude IRR of GBS for PEH compared with the general population was 6.8 (95% CI 2.5–15.0), and the age-adjusted IRR was 6.9 (95% CI 6.0–8.1) (Table 2).

PEH who had invasive pneumococcal infection were younger than persons with invasive pneumococcal infection among the general population (Table 1). They were also more likely than the general population to have recorded alcohol abuse and be diagnosed with pneumonia but less likely to have recorded diabetes. The most common pneumococcal infection serotypes among PEH after 2010 (the year 13-valent pneumococcal conjugate vaccine was introduced in Alaska) were 31 (19%), 16F (15%), F (a vaccine type) (11%), and 9N (11%), whereas the most common serotypes among persons with invasive pneumococcal infection among the general population were 22F (12%), 7F (a vaccine type) (10%), and type 3 (a vaccine type) (9%) (Figure 1). The crude IRR of invasive pneumococcal disease for PEH compared with the general population was 40.3 (95% CI 31.5–51.0), and the age-adjusted IRR was 36.3 (95% CI 33.0–39.9) (Table 2).

During 2002–2015, an excess of 40 cases of invasive GAS, 4 cases of invasive GBS, and 54 cases of invasive pneumococcal infections per 10,000 person-years were estimated to have occurred within the homeless population in Anchorage (data not shown). Of all invasive GAS cases in Anchorage during the study period, 19.6% occurred within the homeless population, whereas 3% of invasive GBS cases and 15.5% of invasive pneumococcal cases were within the homeless population.

## Discussion

A substantial proportion of the disease burden for invasive GAS, GBS, and pneumococcal disease in Anchorage occurred among PEH. Although the estimated homeless population in 2010 accounted for only 0.4% of the total population, nearly 20% of invasive GAS infections, 3% of invasive GBS infections, and 16% of invasive pneumococcal disease occurred within this population. The risk for invasive GAS infection was 53 times higher, the risk for invasive GBS infection 7 times higher, and the risk for invasive pneumococcal infection 36 times higher among PEH compared with the general population.

Commonly identified risk factors for invasive GAS infection among adults include older age, male sex, exposure to children, household crowding, acute and chronic skin breakdown, immune-compromising conditions, heart disease, diabetes, and intravenous drug use (*24*–*26*), whereas established risk factors for invasive GBS among adults include immune-compromising conditions, heart disease, diabetes, and older age (*27*,*28*). Homelessness has not been previously quantified as a major factor for either type of infection, despite recent outbreaks of GAS among PEH (*3*,*13*,*14*). For invasive pneumococcus infection, risk factors include older age, immune-compromising conditions, alcohol use, high body mass index, and cigarette smoking (*29*–*32*). Although the overall incidence of invasive pneumococcal disease was higher in this study than the study in Toronto, Ontario (601 infections/100,000 person-years in Anchorage vs. 273 infections/100,000 person-years in Toronto), the IRR estimates were similar (adjusted IRR of 36 in Anchorage vs. crude IRR of 30 in Toronto) (*16*). The number of cases of invasive GBS in the homeless population over the study period was small, and the IRR was also smaller than for invasive GAS and pneumococcal infections. This finding might reflect a difference in transmission pathways and risk factors between invasive GAS, GBS, and pneumococcal infections.

For all 3 invasive streptococcal diseases, PEH were more likely than the general population to have alcohol abuse recorded but less likely to have diabetes recorded. These differences might reflect either actual differences in invasive streptococcal disease risk factors for PEH compared with the general population or differences in distribution of these factors among each source population. For example, the difference in recorded alcohol abuse might reflect higher alcohol abuse among PEH than the general population or an elevated risk for invasive disease as a result of alcohol abuse. The difference in recorded diabetes diagnoses could reflect a truly lower prevalence of diabetes among PEH with invasive disease or a lack of access to care among PEH (and therefore a lack of diagnoses) compared with the general population.

**Figure 2:**
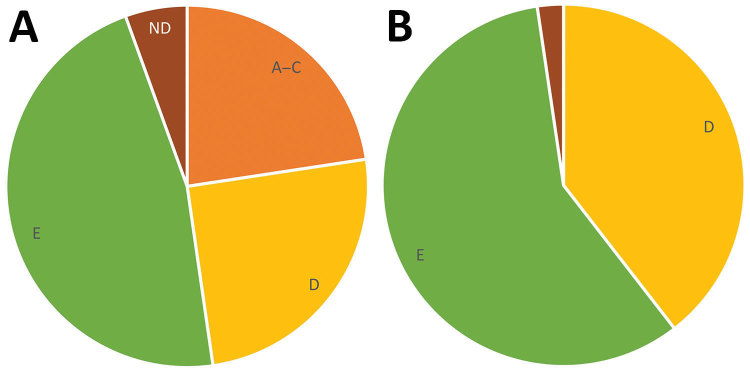
Group A *Streptococcus emm* pattern types among the general population (A) compared with persons experiencing homelessness (B), Anchorage, Alaska, 2002–2015. ND, not determined.

Although GAS molecular types *emm*49 and *emm*82 were common among both PEH and the general population with invasive GAS, we observed some notable differences in *emm* distribution (Figure 1). For example, no cases of *emm*1 infection were identified among PEH, even though it was the most commonly identified *emm* type among Anchorage general population residents with invasive GAS infection in this study. Conversely, a higher proportion of infections among PEH were *emm*91 than among invasive GAS infections in the general population. These differences in *emm-*type distribution demonstrate a larger trend in *emm*-type pattern distribution (Figure 2). Among PEH, no pattern A–C strains were identified, whereas a large proportion of pattern D strains were detected. These type patterns have been associated with tissue tropism (*33*); pattern D strains tend to cause skin infection. This trend suggests that skin breakdown and skin-to-skin transmission could be more important risk factors for invasive GAS disease among PEH than among the general population in Anchorage, which also aligns with the differences in clinical diagnoses between the 2 groups.

As with GAS, the most common serotypes of pneumococcal isolates in persons with invasive pneumococcal infection were not the same for PEH and persons in the general population, although 7F (a 13-valent pneumococcal conjugate vaccine type) was commonly identified in both populations (Figure 1). These differences in distribution could also be a result of social contact patterns among PEH that have low overlap with the general population.

Quantifying the number of PEH and the number of cases of disease in the homeless population is complicated by several factors. First, whether a person is experiencing homelessness could be underestimated in the medical records. To evaluate the extent of underestimation, we conducted a small analysis of the sensitivity of capture of homelessness in the context of a 2016 outbreak of invasive *emm*26.3 GAS infections in Anchorage (*3*). In this outbreak, 24 cases of *emm*26.3 that were identified through the surveillance system were independently evaluated by using chart reviews and interviews; 22 of these were determined to have occurred in PEH. Of these, 18 were captured as homeless in the standard surveillance chart review form used in our study, yielding a sensitivity of 82%. A second possible limitation is that the homeless population could be undercounted by PIT. However, even if the actual size of the homeless population were 3 times larger than estimated, the IRRs for invasive GAS, GBS, and pneumococcal disease comparing PEH to the general population would decrease proportionately but remain large and statistically significant (an adjusted IRR of 18 for GAS, 2 for GBS, and 12 for pneumococcus). 

In addition, we are not able to assess the underlying risk factor distributions in the well population. Therefore, comparing the characteristics of cases in surveillance data limits our ability to assess the difference in risk factors for disease between PEH and the general population. Finally, the health-related causes and outcomes of homelessness are complex. This analysis does not isolate the effect of lacking housing from the myriad conditions that are integrated with experiencing homelessness. The effect of not having housing could lead to exposure to weather, lack of access to hygiene resources, spending time in crowded facilities, and worsening of underlying chronic illnesses, each of which could increase the transmission of invasive streptococcal disease. However, factors such as injection drug use, alcohol use, and unmanaged chronic diseases can also lead to homelessness and are independent risk factors for invasive streptococcal disease. In this analysis, we are unable to estimate how much of the increased risk for invasive streptococcal infection is a result of lacking housing or a result of the factors that led to the lack of housing. Despite these limitations, the health disparities between PEH and the general population indicate that targeted resources could prevent invasive GAS, GBS, and pneumococcal disease, regardless of the ultimate origin of risk.

In 2016, an estimated 1.42 million persons in the United States used an emergency shelter or transitional housing at some point during the year (*34*). According to our analysis, this population is at an increased risk for invasive streptococcal disease, especially invasive GAS and invasive pneumococcal disease. Promoting infection control in shelters, increasing the availability of pneumococcal vaccine, and improving monitoring of infections in homeless populations could improve the health of this vulnerable group.
